# Heterologous saRNA Prime, DNA Dual-Antigen Boost SARS-CoV-2 Vaccination Elicits Robust Cellular Immunogenicity and Cross-Variant Neutralizing Antibodies

**DOI:** 10.3389/fimmu.2022.910136

**Published:** 2022-07-15

**Authors:** Adrian Rice, Mohit Verma, Emily Voigt, Peter Battisti, Sam Beaver, Sierra Reed, Kyle Dinkins, Shivani Mody, Lise Zakin, Shiho Tanaka, Brett Morimoto, C. Anders Olson, Elizabeth Gabitzsch, Jeffrey T. Safrit, Patricia Spilman, Corey Casper, Patrick Soon-Shiong

**Affiliations:** ^1^ ImmunityBio, Inc., Culver City, CA, United States; ^2^ Access to Advanced Health Institute (AAHI), Seattle, WA, United States; ^3^ Departments of Medicine and Global Health, University of Washington, Seattle, WA, United States

**Keywords:** self-amplifying RNA, DNA, vaccine, dual antigen, heterologous, spike, nucleocapsid

## Abstract

We assessed if immune responses are enhanced in CD-1 mice by heterologous vaccination with two different nucleic acid-based COVID-19 vaccines: a next-generation human adenovirus serotype 5 (hAd5)-vectored dual-antigen spike (S) and nucleocapsid (N) vaccine (AdS+N) and a self-amplifying and -adjuvanted S RNA vaccine (AAHI-SC2) delivered by a nanostructured lipid carrier. The AdS+N vaccine encodes S modified with a fusion motif to increase cell-surface expression and an N antigen modified with an Enhanced T-cell Stimulation Domain (N-ETSD) to direct N to the endosomal/lysosomal compartment and increase MHC class I and II stimulation potential. The S sequence in the AAHI-SC2 vaccine comprises the D614G mutation, two prolines to stabilize S in the prefusion conformation, and 3 glutamines in the furin cleavage region to confer protease resistance. CD-1 mice received vaccination by homologous and heterologous prime > boost combinations. Humoral responses to S were the highest with any regimen that included the AAHI-SC2 vaccine, and IgG bound to wild type and Delta (B.1.617.2) variant S1 at similar levels. An AAHI-SC2 prime followed by an AdS+N boost particularly enhanced CD4+ and CD8+ T-cell responses to both wild type and Delta S peptides relative to all other vaccine regimens. Sera from mice receiving AAHI-SC2 homologous or heterologous vaccination were found to be highly neutralizing for all pseudovirus strains tested: Wuhan, Beta, Delta, and Omicron strains. The findings here, taken in consideration with the availability of both vaccines in thermostable formulations, support the testing of heterologous vaccination by an AAHI-SC2 > AdS+N regimen in animal models of SARS-CoV-2 infection to assess its potential to provide increased protection against emerging SARS-CoV-2 variants particularly in regions of the world where the need for cold-chain storage has limited the distribution of other vaccines.

## Introduction

Impressive efforts of the scientific and pharmaceutical community have resulted in the design, testing and successful deployment of several COVID-19 vaccines that have shown high levels of efficacy (1–5). Nonetheless, SARS-CoV-2 viral variants have continued to emerge and spread throughout the globe – most recently the highly transmissible Omicron variant (6) – pointing to the need for delivery of vaccines to populations that are currently underserved.

To address the need for a vaccine regimen that would be highly efficacious against predominating and emerging variants as well as distributable in currently underserved areas, we previously developed a next-generation human adenovirus serotype 5 (hAd5)-vectored dual-antigen spike (S) plus nucleocapsid (N) vaccine (AdS+N) (7, 8) to leverage the resilience of cell-mediated immunity against variants. This vaccine, encoding Wuhan strain or ‘wild type’ (wt) SARS-CoV-2 S and modified with a fusion sequence (S-Fusion) to enhance cell-surface expression (7, 8), as well as N modified with an Enhanced T-cell Stimulation Domain (N-ETSD) (9) for increased MHC class I and II stimulation (10–12), has been shown to elicit humoral and T-cell responses in mice (8), non-human primates (NHP) (7), and participants in Phase 1b trials (9). The AdS+N vaccine given as a subcutaneous (SC) prime with two oral boosts protected NHP from SARS-CoV-2 infection (7), and a single prime vaccination of clinical trial participants generated T-cell responses that were sustained against a series of variant S peptide sequences, including those for the B.1.351, B.1.1.7, P.1, and B.1.426 variants (9).

Despite the promising findings with the AdS+N vaccine candidate, we wish to continue to investigate vaccine regimens with the potential to maximize immune responses – both humoral and cellular. One such approach is by heterologous vaccination utilizing multiple nucleic acid-based vaccine platforms, such as ImmunityBio’s hAd5-vectored DNA vaccine and the Access to Advanced Health Institute’s (AAHI) RNA-based vaccine (13). Heterologous vaccination using vaccine constructs expressing the same or different antigens vectored by different platforms has previously been reported to significantly increase immune responses (14–16), and specifically for COVID-19 vaccines, heterologous prime-boost regimens including the available mRNA and adenovirus-based vaccines elicit humoral and cellular responses in human subjects that are at least as good as or better than homologous vaccination (17–20).

To assess the potential for enhanced immune responses by heterologous vaccination, we tested prime > boost combinations of the AdS+N vaccine with a self-amplifying and self-adjuvanted S(wt) RNA-based vaccine (AAHI-SC2) delivered in a nanostructured lipid carrier (NLC) (21, 22) that has recently been reported to elicit robust, virus-neutralizing humoral responses, establishment of long-lived antibody-secreting plasma cell populations, and polyfunctional CD4+ and CD8+ T-cell responses after both prime and prime-boost regimens in C57BL/6 mice (13). The NLC stabilizes the self-amplifying RNA (23–25) and delivers it to cells, where the vaccine RNA is then amplified and S protein is expressed. The S sequence in the AAHI-SC2 vaccine comprises a codon-optimized sequence with the D614G mutation (26) that increases SARS-CoV-2 susceptibility to neutralization (27), a diproline modification to stabilize S in the pre-fusion conformation that increases antigenicity (28), and a tri-glutamine (3Q) repeat in the furin cleavage region to render it protease resistant (29).

In this work, the two aforementioned vaccines were tested by homologous and heterologous AdS+N > AAHI-SC2 and AAHI-SC2 > AdS+N prime > boost regimens. The findings reported here support our hypothesis that heterologous vaccination with the AAHI-SC2 and AdS+N vaccines enhances immune responses, particularly T-cell responses.

## Methods

### The AdS+N and AAHI-SC2 Vaccines

For studies here, the next generation hAd5 [E1-, E2b-, E3-] vector was used to create the viral vaccine candidate construct (7). This hAd5 [E1-, E2b-, E3-] vector is primarily distinguished from other first-generation [E1-, E3-] recombinant Ad5 platforms (30, 31) by having additional deletions in the early gene 2b (E2b) region that remove the expression of the viral DNA polymerase (pol) and in preterminal protein (pTP) genes, and by its propagation in the E.C7 human cell line (32–35).

The AdS+N vaccine expresses a wild type spike (S) sequence [accession number YP009724390] modified with a proprietary ‘fusion’ linker peptide sequence as well as a wild type nucleocapsid (N) sequence [accession number YP009724397] with an Enhanced T-cell Stimulation Domain (ETSD) signal sequence that directs translated N to the endosomal/lysosomal pathway (9) as described in Gabitzsch *et al.*, 2021 (7).

**Figure 1:**
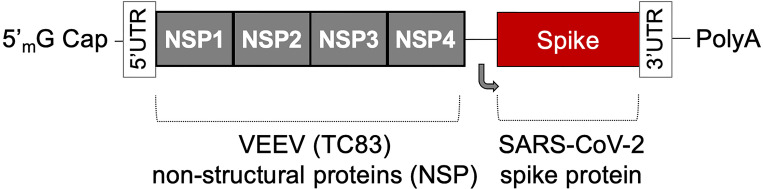
*The saRNA(D614G)-2P-3Q-NLC (AAHI-SC2) vaccine*. The AAHI-SC2 vaccine comprises an saRNA replicon backbone consisting of the non-structural proteins (NSPs) 1-4 derived from the Venezuelan equine encephalitis virus (VEEV) vaccine strain TC-83 and an independent open reading frame under the control of a subgenomic promoter sequence that contains Wuhan sequence S with a diproline (pp) mutation and a QQAQ furin cleavage site sequence.

The AAHI-SC2 vaccine comprises an saRNA replicon composed of an 11.7 kb construct expressing the SARS-CoV-2 S protein, along with the non-structural proteins 1-4 derived from the Venezuelan equine encephalitis virus (VEEV) vaccine strain TC-83 (**Figure 1**). The S RNA sequence is codon-optimized and expresses a protein with the native sequence of the original Wuhan strain plus the dominant D614G mutation, with the prefusion conformation-stabilizing diproline (pp) mutation (consistent with other vaccine antigens) and replacement of the furin cleavage site RRAR sequence with a QQAQ sequence.

The RNA is generated by T7 promoter-mediated *in vitro* transcription using a linearized DNA template. *In vitro* transcription is performed using an in house-optimized protocol (13, 36, 37) using T7 polymerase, RNase inhibitor, and pyrophosphatase enzymes. The DNA plasmid is digested with DNase I, and the RNA is capped by vaccinia capping enzyme, guanosine triphosphate, and S-adenosyl-methionine. RNA is then purified from the transcription and capping reaction components by chromatography using a CaptoCore 700 resin (GE Healthcare) followed by diafiltration and concentration using tangential flow filtration into 10 mM Tris buffer. The RNA material is terminally filtered with a 0.22 μm polyethersulfone filter and stored at -80°C until use.

The RNA-delivering NLC is comprised of particles with a hybrid liquid and solid oil core, providing colloidal stability (21), surrounded by non-ionic hydrophobic and hydrophilic surfactants to help maintain a stable nanoparticle droplet and the cationic lipid DOTAP to provide positive charge for electrostatic binding with RNA. This RNA binding on the surface of the nanoparticles protects the RNA from RNase degradation and allows effective delivery to cells.

NLC is manufactured by mixing the lipids in an oil phase, dissolving the Tween 80 in citrate buffer aqueous phase, and homogenizing the two phases by micro-fluidization. The resulting emulsion is sterile-filtered and vialed until dilution in a sucrose-citrate solution and complexing with vaccine saRNA.

### Murine Immunization and Blood/Tissue Collection

**Figure 2:**
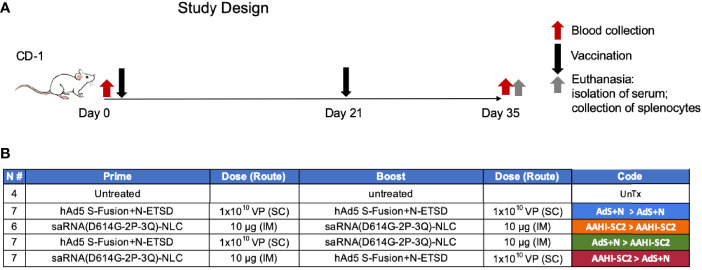
*Study design and vaccine description*. **(A)** CD-1 mice received prime vaccination on Day 0 after blood collection and boost vaccination on Day 21; mice were euthanized and tissues/blood collected on Day 35. **(B)** The various combinations of prime > boost are shown, including: AdS+N homologous; saRNA(D614G-2P-3Q)-NLC (AAHI-SC2) homologous; AdS+N prime, AAHI-SC2 boost; and AAHI-SC2 prime, AdS+N boost. Untreated mice were used as controls. All groups were n = 7 with the exception of untreated n = 4 and AAHI-SC2 homologous n = 6. The color code for each group is shown.

The design of vaccination study performed using CD-1 mice is shown in **Figure 2**.

All *in vivo* experiments described were carried out at the Omeros Inc. vivarium (Seattle, WA) in strict accordance with good animal practice according to NIH recommendations. All procedures for animal use were done under an animal use protocol (#19-08) approved by the IACUC at Omeros, Inc. (Seattle, WA, USA).

CD-1 female mice (Charles River Laboratories) 6-8 weeks of age were used for immunological studies. The adenovirus-vectored vaccines were administered by subcutaneous (SC) injections at 1x10e^10^ viral particles (VP) in 50 µL ARM buffer (20 mM Tris pH 8.0, 25 mM NaCl, with 2.5% glycerol). The AAHI-SC2 vaccine was administered intramuscularly (IM) in 10% sucrose, 5 mM sodium citrate solution at a dose of 10 μg.

On the final day of each study, blood was collected submandibularly from isoflurane-anesthetized mice, and sera were isolated using a microtainer tube. Mice were then euthanized for collection of spleens. Spleens were placed in 5 mL of sterile media (RPMI/HEPES/Pen/Strep/10% FBS). Splenocytes were isolated (38) within 2 hours of collection and used fresh or cryopreserved for later analysis.

### Intracellular Cytokine Stimulation

ICS assays were performed using 10^6^ live splenocytes per well in 96-well U-bottom plates. Splenocytes in RPMI media supplemented with 10% FBS were stimulated by the addition of pools of overlapping peptides spanning the SARS-CoV-2 S protein (both wild type Wuhan strain, wt, or Delta sequence) or N antigens at 1-2 μg/mL/peptide for 6 h at 37°C in 5% CO_2_, with protein transport inhibitor, GolgiStop (BD) added two hours after initiation of incubation. The S peptide pool (wild type, JPT Cat #PM-WCPV-S-1; Delta, JPT cat# PM-SARS2-SMUT06-1) is a total of 315 spike peptides split into two pools, S1 and S2, comprised of 158 and 157 peptides each. The N peptide pool (JPT; Cat # PM-WCPV-NCAP-1) was also used to stimulate cells. A SIV-Nef peptide pool (BEI Resources) was used as an off-target negative control. Stimulated splenocytes were then stained with a fixable cell viability stain (eBioscience™ Fixable Viability Dye eFluor™ 506 Cat# 65-0866-14) followed by the lymphocyte surface markers CD8β and CD4, fixed with CytoFix (BD), permeabilized, and stained for intracellular accumulation of interferon-gamma (IFN-γ), tumor necrosis factor-alpha (TNF-α), and interleukin-2 (IL-2). Fluorescent-conjugated anti-mouse antibodies used for labeling included CD8β antibody (clone H35-17.2, ThermoFisher), CD4 (clone RM4-5, BD), IFN-γ (clone XMG1.2, BD), TNF-α (clone MP6-XT22, BD) and IL-2 (clone JES6-5H4; BD), and staining was performed in the presence of unlabeled anti-CD16/CD32 antibody (clone 2.4G2; BD). Flow cytometry was performed using a Beckman-Coulter Cytoflex S flow cytometer and analyzed using Flowjo software.

### ELISpot Assay

ELISpot assays were used to detect cytokines secreted by splenocytes from inoculated mice. Fresh splenocytes were used on the same day as harvest, and cryopreserved splenocytes containing lymphocytes were used on the day of thawing. The cells (2-4 x 10^5^ cells per well of a 96-well plate) were added to the ELISpot plate containing an immobilized primary antibody to either IFN-γ or IL-4 (BD Cat# 551881 and BD Cat# 551878, respectively), and were exposed to various stimuli (e.g. control peptides SIV and ConA, S-WT and N peptides pools – see catalog numbers above) at a concentration of 1-2 μg/mL peptide pools for 36-40 hours. After aspiration and washing to remove cells and media, extracellular cytokines were detected by a biotin-conjugated secondary antibody to either IFN-γ or IL-4 (BD Cat# 551881 and BD Cat# 551878, respectively), followed by a streptavidin/horseradish peroxidase conjugate (BD Cat# 557630) to detect the biotin-conjugated secondary antibody. The number of spots per well, or per 2-4 x 10^5^ cells, was counted using an ELISpot plate reader. Quantification of Th1/Th2 bias was calculated by dividing the IFN-γ spot forming cells (SFC) per million splenocytes with the IL-4 SFC per million splenocytes for each animal.

### ELISA for Detection of Antibodies

For IgG antibody detection in inoculated mouse sera and lung homogenates, ELISAs for spike-binding (including S1 Delta) and nucleocapsid-binding IgG and IgG subclass (IgG1, IgG2a, IgG2b, and IgG3 antibodies were used. A microtiter plate was coated overnight with 100 ng of either purified recombinant SARS-CoV-2 S-FTD (FL S with fibritin trimerization domain, constructed and purified in-house by ImmunityBio), purified recombinant Spike S1 domain (S1(wt)) (Sino; Cat # 40591-V08B1), purified recombinant Delta variant Spike S1 domain (S1(Delta)) (Sino; Cat # 40591-V08H23), or purified recombinant SARS-CoV-2 nucleocapsid (N) protein (Sino; Cat # 40588-V08B) in 100 µL of coating buffer (0.05 M Carbonate Buffer, pH 9.6). The wells were washed three times with 250 µL PBS containing 1% Tween 20 (PBST) to remove unbound protein, and the plate was blocked for 60 minutes at room temperature with 250 µL PBST. After blocking, the wells were washed with PBST, 100 μL of either diluted serum or diluted lung homogenate samples was added to each well, and samples were incubated for 60 minutes at room temperature. After incubation, the wells were washed with PBST and 100 μL of a 1/5000 dilution of anti-mouse IgG2a HRP (GE Health Care; Cat # NA9310V), anti-mouse IgG2b HRP (Sigma; Cat # SAB3701171), anti-mouse IgG_2a_ HRP (Sigma; Cat # SAB3701178), anti-mouse IgG_2b_ HRP (Sigma; catalog# SAB3701185), or anti-mouse IgG3 HRP conjugated antibody (Sigma; Cat # SAB3701192), (Sigma: Cat #SAB3701192) was added to wells. For positive controls, 100 μL of a 1/5000 dilution of rabbit anti-N IgG Ab or 100 μL of a 1/25 dilution of mouse anti-S serum (from mice immunized with purified S antigen in adjuvant) were added to appropriate wells. After incubation at room temperature for 1 hour, the wells were washed with PBST and incubated with 200 μL o-phenylenediamine-dihydrochloride (OPD substrate, Thermo Scientific Cat # A34006) until appropriate color development. The color reaction was stopped with addition of 50 μL 10% phosphoric acid solution (Fisher Cat # A260-500) in water, and the absorbance at 490 nm was determined using a microplate reader (SoftMax Pro, Molecular Devices).

### Calculation of Relative ng Amounts of Antibodies and the Th1/Th2 IgG Subclass Bias

A standard curve of IgG for OD vs. ng mouse IgG was generated using purified mouse IgG (Sigma Cat #15381); absorbance values from this standard curve were used to convert sample absorbance signals into mass equivalents for both anti-S and anti-N antibodies. Using these values, we calculated the geometric mean value for S- and N-specific IgG per milliliter of serum induced by vaccination. These values were also used to quantify the Th1/Th2 bias for the humoral responses by dividing the sum total of Th1 biased antigen-specific IgG subclasses (IgG2a, IgG2b and IgG3) with the total Th2 indicative IgG1, for each mouse. For mice that lacked anti-S and/or anti-N specific IgG responses, Th1/Th2 ratio was not calculated. Some responses, particularly for anti-N responses in IgG2a and IgG2b (both Th1 biased subclasses), were above the limit of quantification with OD values higher than those observed in the standard curve. These data points were therefore reduced to values within the standard curve, and thus the reported Th1/Th2 bias is lower than would otherwise be reported.

### Endpoint titers

Serial dilutions were prepared from each serum sample, with dilution factors ranging from 400 to 6,553,600 in 4-fold steps. These dilution series were characterized by whole IgG ELISA assays against both recombinant S1(wt) and recombinant S1(Delta), as described above. Half maximal response values (Ab_50_) were calculated by non-linear least squares fit analysis on the values for each dilution series against each recombinant S1 in GraphPad Prism. Serum samples from mice without anti-S responses were removed from Ab_50_, μg IgG/mL sera, and endpoint titer analyses and reported as N/D on the graphs. Endpoint titers were defined as the last dilution with an absorbance value at least 3 standard deviations higher than the standard deviation of all readings from serum of untreated animals (n = 32 total negative samples). Quantitative titration values (μg IgG/mL sera) were calculated against a standard curve as described above.

### Pseudovirus Neutralization Assay

SARS-CoV-2 pseudovirus neutralization assays were conducted on immunized mouse serum samples using procedures adapted from Crawford *et al.*, 2020 (39). In brief, lentiviral pseudoviruses expressing SARS-CoV-2 spike protein variants were prepared by co-transfecting HEK293 cells (ATCC CRL-3216) seeded at 4x10^e5^ cells/mL with a plasmid containing a lentiviral backbone expressing luciferase and ZsGreen (BEI Resources NR-52516), plasmids containing lentiviral helper genes (BEI Resources NR-52517, NR-52518, NR-52519), a delta19 cytoplasmic tail-truncated SARS-CoV-2 spike protein expression plasmid (Wuhan strain, B.1.1.7, and B.1.351 spike variant plasmids were a gift from Jesse Bloom of Fred Hutchinson Cancer Research Center; B.1.617.2 Delta and Omicron variant plasmids were a gift from Thomas Peacock of Imperial College London) and Bio-T transfection reagent (Bioland Scientific B0101). The transfection was incubated for 72 hours at 37°C, 5% CO_2_. Pseudovirus stocks were harvested from the cell culture media, (Gibco DMEM + GlutaMAX + 10% FBS) filtered through a 0.2 μm filter, and frozen at -80°C until titering and use.

Mouse serum samples were diluted 1:10 in media (Gibco DMEM + GlutaMAX + 10% FBS) and then serially diluted 1:2 for 11 total dilutions, and incubated for 1 hour at 37°C, 5% CO_2_.with a mixture of 5 μg/mL polybrene (Sigma TR-1003-G) and pseudovirus diluted to a titer that produces 1×10^e8^ total integrated intensity units/mL. The serum-virus mix was then added in duplicate to human Angiotensin-Converting Enzyme 2 expressing HEK293 cells (BEI Resources NR-52511, NIAID, NIH) seeded at 4 x 10^e5^ cells/mL on a 96 well plate.

The plates were incubated at 37°C, 5% CO_2_ for 72 hours. Plates were imaged on a high content fluorescent imager (Molecular Devices ImageXpress Pico) for ZsGreen expression. Total integrated intensity units per well quantified using ImageXpress software (Molecular Devices) was used to calculate % pseudovirus inhibition in each well. Neutralization curves were fit with a four-parameter sigmoidal curve which was used to calculate 50% inhibitory concentration dilution (IC50) values.

### Statistical Analyses and Graph Generation

All statistical analyses were performed and figures and graphs generated using GraphPad Prism software. Data that did not have a normal distribution as determined by a Shapiro-Wilks test were analyzed using a non-parametric Kruskal-Wallis test with Dunn’s *post-hoc* comparison of groups and were graphed as the mean and standard deviation (SD). Data graphed on a log scale were log-normalized, analyzed using one-way ANOVA and Tukey’s comparison of groups, and were graphed as the geometric mean and the geometric SD. Statistical analyses of Endpoint Titers for anti-S1 IgG were performed by assigning a value of 200 – one half the Level of Detection (LOD) of 400 – to the 4 animals with serum values below the LOD. P values for each comparison are listed in **Supplementary Table S1**.

## Results

### The AAHI-SC2 Vaccine Enhances Generation of Anti-S(wt) IgG

**Figure 3:**
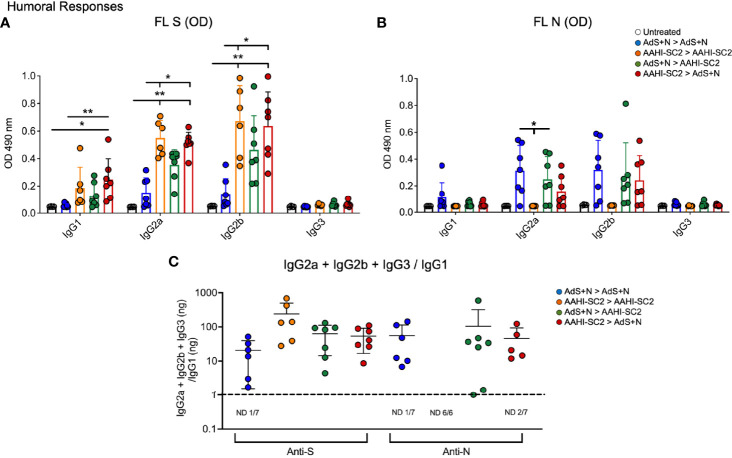
*Anti-full length (FL) spike wild type (Swt) and -nucleocapsid (N) IgG antibody levels in sera show T helper cell 1 (Th1) bias*. **(A)** Levels of anti-FL Swt and **(B)** anti-N IgG1, IgG2a, IgG2b and IgG3 subtypes represented by OD at 490 nm from ELISA of sera are shown. Statistical analyses were performed using a non-parametric Kruskal-Wallis test and Dunn’s *post-hoc* comparison of all groups where *p ≤.05 and **p <.01. In instances of similar significance, the tick marks indicate groups compared to the group without a tick mark; for example in panel A, both the AAHI-SC2 homozygous (orange) and AAHI-SC2 > AdS+N (red) groups showed **p <.01 signifcant increases as compared to the untreated group (clear) for IgG2a. P values are listed in Table 1. The legend in B applies to panels **(A, B)**. All dilutions were 1:400. **(C)** The IgG2a+IgG2b+IgG3/IgG1 ratio calculated using the ng equivalents for each is shown with a dashed line at 1. Values > 1 reflect Th1 bias. The number (n) of animals in which the ratio was not determined due to very low antibody levels is shown below the x-axis for each group. The homologous AAHI-SC2 group did not receive an N antigen. Data graphed as the mean and SD.

Mice that received either AAHI-SC2 homologous or AAHI-SC2 > AdS+N heterologous vaccination had the higher levels of anti-full length S(wt) (FL S) IgG2a and 2b when compared to untreated or AdS+N homologous vaccinated mice, as determined by ELISA OD readouts OD at 490 nm (**Figure 3A**). Only mice receiving the N antigen generated anti-N IgG (also determined by ELISA 490 nm OD readouts at 490 nm); there were no significant differences between the groups that received AdS+N homologous, prime, or boost vaccination (**Figure 3B**). Determination of the IgG2a + IgG2b + IgG3/IgG1 ratio using ng amounts calculated from the OD reading (see *Methods*) revealed responses were highly T helper cell 1 (Th1)-biased, with calculated values being one or greater (**Figure 3C**).

### Humoral Responses Against Wildtype and Delta S1 Were Similar in all AAHI-SC2 Groups

To assess serum antibody production specific for Delta B.1.617.2 variant as compared to wild type (wt) S, ELISAs were performed using either the wt or B.1.617.2 sequence S1 domain of S, which contains the RBD.

**Figure 4:**
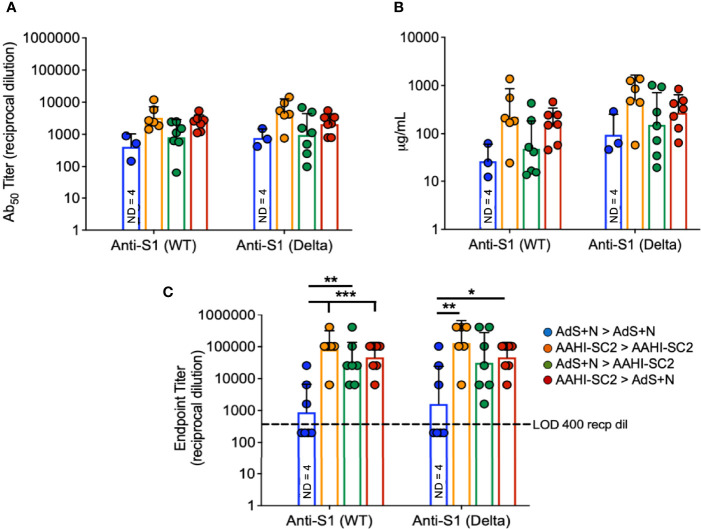
*Wildtype and B.1.617.2 ‘Delta’ S1-specific IgG endpoint titers*. Levels of anti-S1(wt) and -Delta S1 IgG are shown by **(A)** Ab_50_ reciprocal dilution, **(B)** μg/mL sera, and **(C)** endpoint titer reciprocal dilution. Values were below the level of detection in 4 of 7 AdS+N homologous group mice. Statistical analyses were performed on log-normalized data using one-way ANOVA and Tukey’s *post-hoc* comparison of all groups for anti-S1 (WT) or -S1 (Delta) where *p ≤.05, **p <.01 and ***p <.001; in **(C)**, sera without detectable levels of anti-S1 IgG were assigned a value of 200, one-half the Limit of Detection (LOD) of 400. In instances of similar significance, the tick marks indicate groups compared to the group without a tick mark; p values are listed in Table 1. Data graphed as the geometric mean and geometric SD. The legend in C applies to all figure panels.

There were no statistical differences among groups that received the AAHI-SC2 vaccine in any regimen for anti-S1(wt) or -S1(Delta) Ab_50_ or μg IgG/mL (**Figures 4A, B**, respectively); statistical comparison of the AdS+N homologous group to other groups was not performed in **Figure 4A** or **B** because 4 of 7 values were below the LOD. For the endpoint titer reciprocal dilution (**Figure 4C**), AdS+N sera below the LOD were assigned the value of 200 (half the LOD of 400) to allow statistical analysis. Anti-S1(wt) IgG responses were higher for AAHI-SC2 homologous and AAHI-SC2 > AdS+N group mice compared with AdS+N homologous vaccination. Anti-S1(Delta) IgG responses were significantly higher in animals in the AAHI-SC2 homologous group versus the AdS+N homologous group. Other comparisons were not significant due to variation among individual mice.

### An AdS+N Boost After AAHI-SC2 Prime Vaccination Enhances CD4+ and CD8+ T-Cell Responses to S Peptides

**Figure 5:**
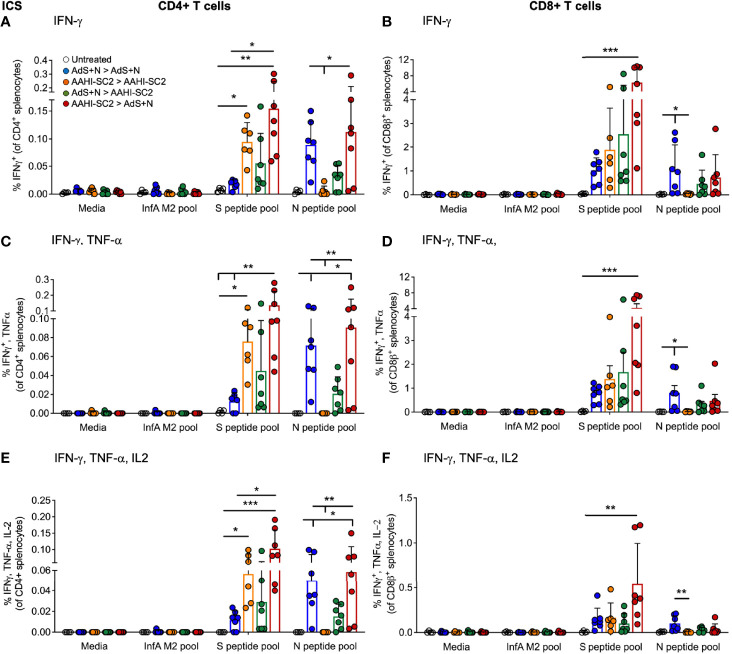
*CD4+ and CD8+ T cell intracellular cytokine staining (ICS) in response to S(wt) and N peptides*. **(A, B)** ICS for interferon-γ (IFN-γ), **(C, D)** IFN-γ and tumor necrosis factor-α (TNF-α), and **(E, F)** IFN-γ, TNF-α and interleukin-2 (IL-2) are shown for CD4+ and CD8+ T cells, respectively. Statistical analyses performed using a non-parametric Kruskal-Wallis test and Dunn’s *post-hoc* comparison of all groups to all other groups where *p ≤.05, **p <.01, and ***p <.001. In instances of similar significance, the tick marks indicate groups compared to the group without a tick mark; p values are listed in Table 1. Data graphed as the mean and SD. The legend in A applies to all figure panels.

Significantly higher percentages of CD4+ T-cells secreting IFN-γ alone, IFN-γ and tumor necrosis factor-α (TNF-α), or IFN-γ, TNF-α, and interleukin-2 (IL-2) as detected by intracellular cytokine staining (ICS) in response to S(wt) peptides were detected in the AAHI-SC2 > AdS+N - but not AdS+N > AAHI-SC2 - group mice as compared to the untreated and AdS+N homologous group (**Figures 5A, C, E**). Although mean values for the AdS+N > AAHI-SC2 group were lower than those for the AAHI-SC2 > AdS+N group, the differences were not statistical significant due to individual variation among mice.

Only cytokine production by CD8+T cells from AAHI-SC2 > AdS+N group mice was significantly greater than the untreated group (**Figures 5B, D, F**), and the level of significance was greater than that observed for CD4+ T cells (**Figures 5A, C, E**).

Only T cells from mice receiving vaccination regimens that included delivery of the N antigen by the AdS+N vaccine produced cytokines in response to N peptide stimulation. For CD4+ T cells, IFN-γ (IFN-γ) production was significantly greater for AdS+N homologous and AAHI-SC2 > AdS+N groups (but not the AdS+N > AAHI-SC2 group) compared to the AAHI-SC2 homologous group (**Figure 5A**), and IFN-γ, tumor necrosis factor-α (TNF-α) as well as IFN-γ, TNF-α, and interleukin-1 (IL-2) production were greater for the same two groups as compared to either the untreated or AAHI-SC2 homologous groups (**Figures 5C, E**, respectively). For CD8+ T cells, only the AdS+N homologous group had significantly greater cytokine production than the groups that did not receive N (**Figures 5B, D, F**).

### CD4+ and CD8+ T-Cell Production of IFN-γ Was Similar in Response to Either S(wt) or S(Delta) Peptides

**Figure 6:**
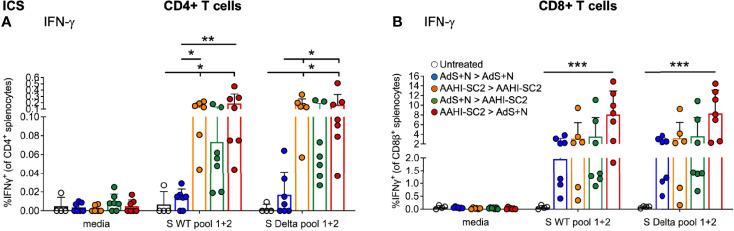
*CD4+ and CD8+ T-cell responses to S(wt) and S(Delta) peptides are similar*. Both CD4+ **(A)** and CD8+ **(B)** T cells show similar levels of interferon-γ (IFN-γ) production in ICS in response to either S(wt) or S(Delta) sequence peptides. For both T-cell types, the greatest responses were seen with AAHI-SC2 > AdS+N vaccination. Statistical analyses performed using a non-parametric Kruskal-Wallis test with Dunn’s comparison of groups where *p ≤.05, **p <.01, and ***p <.001. In instances of similar significance, the tick marks indicate groups compared to the group without a tick mark; p values are listed in Table 1. Data graphed as the mean and SD. The legend in B applies to both figure panels.

CD4+ and CD8+ T cells show similar levels of IFN-γ production by ICS in response to either S(wt) or S(Delta) sequence peptides (**Figures 6A, B**, respectively). Patterns of CD4+ and CD8+ T-cell stimulation by S protein peptides between the vaccination regimens were also similar between the S(wt) and S(Delta) peptides. Compared to the untreated control, the increase in IFN-γ production was again the highest for the AAHI-SC2 > AdS+N group for both CD4+ and CD8+ T cells, in response to either S(wt) or S(Delta) peptides.

### Numbers of IFN-γ-Secreting Splenocytes in Response to S Peptides Were the Highest From Mice Receiving AAHI-SC2 > AdS+N Heterologous Vaccination

**Figure 7:**
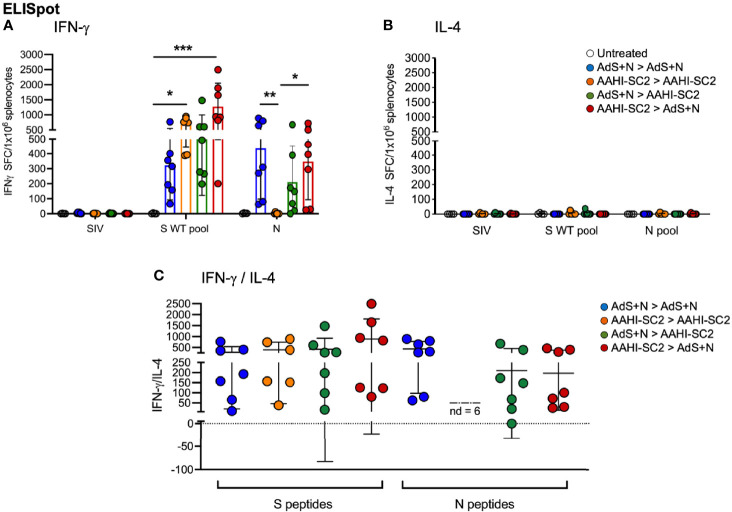
*Heterologous vaccination increases T-cell cytokine secretion in ELISpot*. **(A)** Numbers of interferon-γ (IFN-γ) and **(B)** interleukin-4 (IL-4) secreting T cells in response to S WT and N peptides pools. The legend in B applies to panels A and B. **(C)** The IFN-γ/IL-4 ratio; value of 1 indicated by dashed line. The ratio was not determined (ND) for animals with very low IL-4 secretion. Statistical analyses performed using a non-parametric Kruskal Wallis test and Dunn’s *post-hoc* comparison of all groups to all other groups where *p ≤.05, **p <.01 and ***p<.001. Data graphed as the mean and SD.

As shown in **Figure 7A**, ELISpot detection of cytokine secreting cells in response to S peptide stimulation revealed that animals receiving either homologous AAHI-SC2 or heterologous AAHI-SC2 > AdS+N vaccination developed significantly higher levels of S peptide-reactive IFN-γ-secreting T cells than untreated group animals; the level of significance was greater with heterologous vaccination. Numbers of IFN-γ-secreting T cells in response to the N peptide pool were similar for AdS+N homologous and AAHI-SC2 > AdS+N groups. T cells from AAHI-SC2 > AAHI-SC2 group animals did not secrete IFN-γ in response to the N peptide pool, as expected, because the AAHI-SC2 vaccine does not deliver the N antigen. There was some skew seen for data in **Figure 7A**, with values for S WT/N of untreated = 2.0/0.0, AdS+N > AdS+N = 1.27/0.27, AAHI-SC2 > AAHI-SC2 = -0.53/2.45, AdS+N > AAHI-SC2 = 1.89/1.4, and AAHI-SC2 > AdS+N = 0.35/-0118. We note these are outbred mice with variance in MHC haplotype and variable T-cell data not unexpected.

Reflecting the Th1 bias of T-cell responses, induction of interleukin-4 (IL-4) secreting T cells was low for all animals in all groups (**Figure 7B**); therefore the IFN-γ/IL-4 ratio was above 1 for all animals for which the ratio could be calculated, with the exception of 1 animal in the AdS+N > AAHI-SC2 group in response to N (**Figure 7C**).

### Sera From Mice Receiving the AAHI-SC2 Vaccine Neutralize SARS-CoV-2 Wuhan, Delta, Beta and Omicron Pseudoviruses

**Figure 8:**
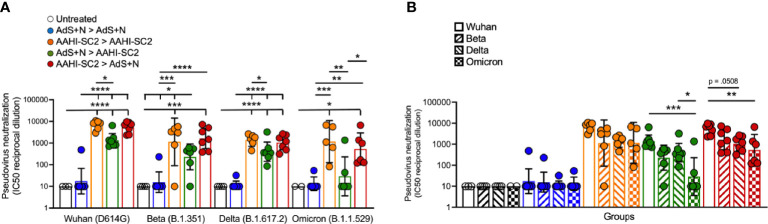
*Sera from AAHI-SC2 > AdS+N heterologously vaccinated mice neutralize Wuhan, Delta, Beta, and Omicron SARS-CoV-2 pseudoviruses*. **(A)** IC50 reciprocal dilution for pseudovirus neutralization grouped by pseudovirus variant assay is shown. Statistical differences are shown for comparison of each vaccinated group for a specific variant (not between variants). **(B)** IC50 reciprocal dilution for neutralization of all strains/variants tested compared for each group is shown. The color code legend in **(A)** applies also to **(B)**. Statistical comparison of IC50 values for untreated and AdS+N homologous group mice with values < the LOD was not performed. Statistical analyses were performed on log-normalized data using one-way ANOVA and Tukey’s *post-hoc* comparison where *p ≤.05, **p <.01, ***p <.001, and ****p <.0001. In instances of similar significance, the tick marks indicate groups compared to the group without a tick mark; p values are listed in Table 1. Data graphed as the geometric mean and the geometric SD.

As represented in **Figure 8A**, sera from AAHI-SC2 homologous and AAHI-SC2 > AdS+N heterologous group mice showed the highest neutralization capability against the four SARS-CoV-2 lentiviral pseudoviruses: Wuhan (D614G), Beta (B.1.351), Delta (B.1.617.2), and Omicron (B.1.1.529) variants. Neutralizing antibody titers were significantly higher than for sera from untreated and AdS+N > AdS+N group mice.

Comparison of SARS-CoV-2 variant neutralizing antibody titers between groups (**Figure 8B**) demonstrate that sera from AAHI-SC2 homologous, AdS+N > AAHI-SC2 and AAHI-SC2 > AdS+N heterologous vaccinated mice all have high Wuhan-strain neutralization capacity. There were no significant differences in the capability of sera from AAHI-SC2 homologous vaccinated mice to neutralize the 4 strains tested, but sera from both heterologously vaccinated groups showed a greater capability to neutralize the Wuhan strain than the Omicron strain.

## Discussion

The immune responses observed in the present study support our hypothesis, and that of others, that heterologous vaccination provides an opportunity for increased humoral and cell-mediated responses to vaccination. These results are consistent with recently-published data reporting enhanced antibody and T-cell responses in patients who received heterologous vaccination with the currently available COVID-19 vaccines (17–20).

Perhaps the most intriguing finding in the present study was that the increases in S-specific CD4+ and CD8+ T-cell responses from heterologous AAHI-SC2 > AdS+N group mice as compared to untreated mice had the highest level of significance, with greater than 5% of CD8+ T cells accumulating both IFN-γ and TNF-α in response to S peptides, on average. Enhancement of T-cell responses when an adenovirus vaccine is used as a boost for an RNA vaccine prime is consistent with both Liu *et al.* (18), who assessed humoral and cellular responses in participants who received ChAdOx or BNT162b2 in various heterologous and homologous prime-boost combinations and concluded the BNT prime > ChAdOx boost regimen resulted in the greatest expansion of vaccine-antigen responsive T cells; and with Atmar *et al.* (20), who found that with various prime > boost regimens with the Ad26.COV2.S and mRNA1273 or BNT162b2 vaccines, heterologous boosting with the Ad26.COV2.S vaccine substantially increased spike-specific CD8+ T cells in the mRNA vaccine-primed recipients.

The enhanced T-cell activity in the AAHI-SC2 > AdS+N group mice was observed in both ICS and ELISpot and for CD8+ T-cells, was seen in response to both wild type and Delta S peptides. Responses of CD4+ T cells to S(wt) and S(Delta) were similar for AAHI-SC2 homologous and AAHI-SC2 > AdS+N heterologous group mice. We hypothesize that because the AAHI-SC2 vaccine elicits the greatest humoral response to S when given in any order – possibly reaching the upper detection limit for our ELISA - it enhances CD4+ T-cell activation as such activation is closely related to humoral/B cell responses. Therefore, CD4+ T-cell activation might be expected to be higher after a boost if there are stronger pre-existing, prime-induced B cell responses, that is, when AAHI-SC2 is the prime. Adenovirus vectors such as that used for the AdS+N vaccine are good at eliciting CD8+ T-cell responses (40), an effect that likely also benefits from more robust pre-existing CD4+ T-cell and B cell responses, a condition that exists most prominently when the AAHI-SC2 vaccine is given as the prime.

Effectively, enhanced CD4+-specific T-helper responses seen with AAHI-SC2 prime dosing might have provided conditions for the enhanced CD8+ specific response upon AdS+N boost. Confirmation of this hypothesis awaits further investigation.

Importantly, all of the vaccination regimens that included the AAHI-SC2 vaccine neutralized SARS-CoV-2 variant pseudoviruses – Wuhan, Beta, Delta, and – for AAHI-SC2 homologous vaccination - the highly transmissible Omicron (BA.1) variant. The heterologous vaccine regimens resulted in lower capability of neutralizing Omicron BA.1 variant, reported to be more resistant to neutralization than the BA.2 variant (41) now displacing BA.1. This neutralization capability reflects the strength of humoral responses to the AAHI-SC2 vaccine and is consistent with reported findings for this vaccine (13). The validity of such pseudovirus-based assay results and their correlation to live virus assays has been reported elsewhere (42, 43). We observed that the geometric mean IC50s for reciprocal dilutions of sera from mice receiving the heterologous AAHI-SC2 > AdS+N regimen were consistently higher than those for AdS+N > AAHI-SC2, and speculate that the AAHI-SC2 as a prime triggers greater B cell priming and development (as compared to AdS+N as the prime) which then results in enhanced recall when the AdS+N boost is delivered.

The lower capability of sera from AdS+N homologously vaccinated mice to neutralize the S-expressing pseudovirus does not necessarily indicate that the predominantly T-cell inducing AdS+N vaccine would not be effective in protecting against SARS-CoV-2 challenge. The pseudovirus assay does not reveal the protection conferred by T-cells and non-neutralizing antibodies against natural infection, which may be enhanced by addition of the N antigen. In fact, we have previously reported that homologous AdS+N prime-boost vaccination of non-human primates confers protection against viral challenge (7). In the *in vivo* viral challenge testing paradigm, cell-mediated immunity - not accessed in the pseudovirus assay that tests sera - conferred by AdS+N vaccination likely plays a key role in protection, as has been reported for natural infection of patients (44–47). Others have reported that combination of S and N increased provided enhanced protection against infection by variants with highly mutated spike in a hamster model (48), and thus, in future studies, we plan to assess protection against infection by SARS-CoV-2 variants *in vivo* by the dual-antigen vaccine as compared to S or N alone.

There were limitations to the study performed, including there being a single time interval between prime and boost tested (21 days) and a single time point for sample collection (35 days). Our goal was to elicit vigorous T-cell responses while also detecting humoral responses, but the relatively short prime-boost interval as well as time to tissue collection may have favored saRNA-induced over adenovirus (AdS+N) generated humoral responses. In addition, a limitation may be that the S antigen in both vaccines is not the Omicron sequence, given that Omicron is currently the predominant variant in many regions. But recent reports suggest Omicron infection does not produce sera that is highly cross-reactive for other Variants of Concern (VOCs) and that a vaccine delivering an Omicron-based spike immunogen is unlikely to be superior to existing vaccines for prime vaccination (49).

The findings here support ongoing study of heterologous vaccination with the AAHI-SC2 and AdS+N vaccines. In our continued efforts, we are designing vaccines with Omicron S sequences and an saRNA vaccine that delivers both an S and N antigen. Further testing in pre-clinical models of SARS-CoV-2 challenge and clinical trials should be conducted to assess the capability of this vaccine regimen to provide increased protection against COVID-19 and SARS-CoV-2 variants by combining the ability of AAHI-SC2 to elicit vigorous humoral responses with AdS+N’s second, highly antigenic N antigen and T-cell response enhancement. In addition to the opportunity for a high level of efficacy, the availability of both the AAHI-SC2 and AdS+N vaccines in thermostable formulations addresses a critical issue in vaccine technology - freedom from cold-chain limitations on distribution - and provides further justification for their continued development.

## Data Availability Statement

The datasets presented in this study are within the manuscript, online at doi: 10.1101/2021.11.29.470440, and available by request.

## Ethics Statement

The animal study was reviewed and approved by the Institutional Animal Care and Use Committee (IACUC) at Omeros, Inc. (Seattle, WA, USA).

## Author Contributions

AR and MV contributed to the study design, co-wrote the manuscript and, with KD and SM, performed the *in vivo* studies and co-analyzed data. EV is co-inventor of the RNA technology used for the AAHI-SC2 saRNA vaccine, contributed to design of the study, co-analyzed data and data interpretation, and edited the manuscript. SB performed the pseudovirus neutralization assay, with the assistance of PB and SR, and edited the manuscript. LZ, CO, ST, and BM contributed to the design, production, and testing of the AdS+N vaccine. EG co-designed the AdS+N vaccine vector. JS contributed to the study design and provided expert immunological/biological insight for interpretation of data. PS analyzed data, generated figures and tables, and wrote the manuscript. CC contributed to the study design and data analysis, and edited the manuscript. PS-S co-designed and developed the AdS+N vaccine, co-conceptualized the study, reviewed all data, and edited the manuscript. All authors contributed to the article and approved the submitted version.

## Funding

The original development of the AAHI-SC2 vaccine was funded by the Infectious Disease Research Institute (IDRI).

## Author Disclaimer

All authors with an ImmunityBio, Inc., affiliation contribution to the design, production or testing of the AdS+N vaccine that may become a commercial product. Emily Voigt is an inventor on a patent related to the RNA vaccine technology.

## Conflict of Interest

Author EV is one of the inventors of the AAHI-SC2 vaccine and all authors with an ImmunityBio, Inc. affiliation are employees of and/or hold shares of ImmunityBio, Inc. stock, which is developing the AdS+N vaccine as a potential product.

The remaining authors declare that the research was conducted in the absence of any commercial or financial relationships that could be constructed as a potential conflict of interest.

This study received funding from ImmunityBio, Inc. The funder had the following involvement with the study: design and manufacturing of the AdS+N vaccine, design and performance of the in vivo study, including tissue collection and analysis; and the writing of the manuscript and decision to publish.

## Publisher’s Note

All claims expressed in this article are solely those of the authors and do not necessarily represent those of their affiliated organizations, or those of the publisher, the editors and the reviewers. Any product that may be evaluated in this article, or claim that may be made by its manufacturer, is not guaranteed or endorsed by the publisher.
