# Supplementary material for: Heterologous saRNA Prime, DNA Dual-Antigen Boost SARS-CoV-2 Vaccination Elicits Robust Cellular Immunogenicity and Cross-Variant Neutralizing Antibodies
Source: Front Immunol. 2022 Jul 15;13:910136. doi: 10.3389/fimmu.2022.910136 (PMC9335885; doi:10.3389/fimmu.2022.910136)
Supplement: Supplementary file 1 [file Table_1.docx]

**Heterologous saRNA Prime, DNA Dual-Antigen Boost SARS-CoV-2 Vaccination Elicits Robust Cellular Immunogenicity and Cross-Variant Neutralizing Antibodies**

Adrian Rice, Mohit Verma, Emily Voigt, Peter Battisti, Sam Beaver, Sierra Reed, Kyle Dinkins, Shivani Mody, Lise Zakin, Shiho Tanaka, Brett Morimoto, C. Anders Olson, Elizabeth Gabitzsch, Jeffrey T. Safrit, Patricia Spilman, Corey Casper, Patrick Soon-Shiong

**Supplementary Table**

**Table S1.** *P values for statistical comparisons.*

| **Table 1. List of p values** | | | | |
| --- | --- | --- | --- | --- |
| **Fig. Panel** | **Parameter** | **Comparison** | **Test; comparison test** | **p value** |
| Fig. 3A | IgG1 | Untx to AAHI-SC2 > AdS+N | Kruskal-Wallis; Dunn's | 0.011 |
| " | " | AdS+N (Ho) to AAHI-SC2 > AdS+N | " | 0.0064 |
| " | IgG2a | Untx to AAHI-SC2 (Ho) | " | 0.0023 |
| " | " | Untx to AAHI-SC2 > AdS+N | " | 0.0019 |
| " | " | AdS+N (Ho) to AAHI-SC2 (Ho) | " | 0.0166 |
| " | " | AdS+N (Ho) to AAHI-SC2 > AdS+N | " | 0.0135 |
| " | IgG2b | Untx to AAHI-SC2 (Ho) | " | 0.0054 |
| " | " | Untx to AAHI-SC2 > AdS+N | " | 0.005 |
| " | " | AdS+N (Ho) to AAHI-SC2 (Ho) | " | 0.0409 |
| " | " | AdS+N (Ho) to AAHI-SC2 > AdS+N | " | 0.0378 |
| Fig. 3B | IgG2a | AdS+N (Ho) to AAHI-SC2 (Ho) | " | 0.0195 |
| " | " | AAHI-SC2 (Ho) to AdS+N > AAHI-SC2 | " | 0.0168 |
| Fig. 4C | Anti-S1 (WT) | AdS+N (Ho) to AAHI-SC2 (Ho) | one-way ANOVA on log-normalized data; Tukey's | 0.0001 |
| " | " | AdS+N (Ho) to AdS+N > AAHI-SC2 | " | 0.0013 |
| " | " | AdS+N to AAHI-SC2 > AdS+N | " | 0.0004 |
| " | Anti-S1 (Delta) | AdS+N (Ho) to AAHI-SC2 (Ho) | " | 0.0035 |
| " | " | AdS+N (Ho) to AAHI-SC2 > AdS+N | " | 0.0226 |
| Fig. 5A | S peptide pool | Untx to AAHI-SC2 (Ho) | Kruskal-Wallis; Dunn's | 0.0178 |
| " | " | Untx to AAHI-SC2 > AdS+N | " | 0.0017 |
| " | " | AdS+N (Ho) to AAHI-SC2 > AdS+N | " | 0.0129 |
| " | N peptide pool | AdS+N (Ho) to AAHI-SC2 (Ho) | " | 0.012 |
| " | " | AAHI-SC2 (Ho) to AAHI-SC2 > AdS+N | " | 0.0177 |
| Fig. 5B | S peptide pool | Untx to AAHI-SC2 > AdS+N | " | 0.0005 |
| " | N peptide pool | Untx to AdS+N (Ho) | " | 0.0378 |
| " | " | AdS+N (Ho) to AAHI-SC2 (Ho) | " | 0.0437 |
| Fig. 5C | S peptide pool | Untx to AAHI-SC2 (Ho) | " | 0.0258 |
| " | " | Untx to AAHI-SC2 > AdS+N | " | 0.0017 |
| " | " | AdS+N (Ho) to AAHI-SC2 > AdS+N | " | 0.0098 |
| " | N peptide pool | Untx to AdS+N (Ho) | " | 0.0168 |
| " | " | Untx to AAHI-SC2 > AdS+N | " | 0.02 |
| " | " | AdS+N (Ho) to AAHI-SC2 (Ho) | " | 0.004 |
| " | " | AAHI-SC2 (Ho) to AAHI-SC2 > AdS+N | " | 0.005 |
| Fig. 5D | S peptide pool | Untx to AAHI-SC2 > AdS+N | " | 0.0006 |
| " | N peptide pool | Untx to AdS+N (Ho) | " | 0.0327 |
| " | " | AdS+N (Ho) to AAHI-SC2 (Ho) | " | 0.0174 |
| Fig. 5E | S peptide pool | Untx to AAHI-SC2 (Ho) | " | 0.0208 |
| " | " | Untx to AAHI-SC2 > AdS+N | " | 0.0009 |
| " | " | AdS+N (Ho) to AAHI-SC2 > AdS+N | " | 0.0106 |
| " | N peptide pool | Untx to AdS+N (Ho) | " | 0.0209 |
| " | " | Untx to AAHI-SC2 > AdS+N | " | 0.076 |
| " | " | AdS+N (Ho) to AAHI-SC2 (Ho) | " | 0.0053 |
| " | " | AAHI-SC2 (Ho) to AAHI-SC2 > AdS+N | " | 0.0042 |
| Fig. 5F | S peptide pool | Untx to AAHI-SC2 > AdS+N | " | 0.0015 |
|  | N peptide pool | AdS+N (Ho) to AAHI-SC2 (Ho) | " | 0.0083 |
| Fig. 6A | S(WT) peptide pool | Untx to AAHI-SC2 (Ho) | " | 0.0221 |
| " | " | Untx to AAHI-SC2 > AdS+N | " | 0.0121 |
| " | " | AdS+N (Ho) to AAHI-SC2 (Ho) | " | 0.0136 |
| " | " | AdS+N (Ho) to AAHI-SC2 > AdS+N | " | 0.006 |
| " | S(delta) peptide pool | Untx to AAHI-SC2 (Ho) | " | 0.0138 |
| " | " | Untx to AAHI-SC2 > AdS+N | " | 0.0195 |
| " | " | AdS+N (Ho) to AAHI-SC2 (Ho) | " | 0.0204 |
| " | " | AdS+N (Ho) to AAHI-SC2 > AdS+N | " | 0.0289 |
| Fig. 6B | S(WT) peptide pool | Untx to AAHI-SC2 > AdS+N | " | 0.001 |
| " | S(delta) peptide pool | Untx to AAHI-SC2 > AdS+N | " | 0.0008 |
| Fig. 7A | S(WT) peptide pool | Untx to AAHI-SC2 (Ho) | " | 0.026 |
| " | " | Untx to AAHI-SC2 > AdS+N | " | 0.001 |
| " | N peptide pool | AdS+N (Ho) to AAHI-SC2 (Ho) | " | 0.0035 |
| " | " | AAHI-SC2 (Ho) to AAHI-SC2 > AdS+N | " | 0.01 |
| Fig. 8A | Wuhan | Untx to AAHI-SC2 (Ho) | one-way ANOVA on log-normalized data; Tukey's | <0.0001 |
| " | " | Untx to AdS+N > AAHI-SC2 | " | <0.0001 |
| " | " | Untx to AAHI-SC2 > AdS+N | " | <0.0001 |
| " | " | AdS+N (Ho) to AAHI-SC2 (Ho) | " | <0.0001 |
| " | " | AdS+N (Ho) to AdS+N > AAHI-SC2 | " | <0.0001 |
| " | " | AdS+N (Ho) to AAHI-SC2 > AdS+N | " | <0.0001 |
| " | " | AAHI-SC2 (Ho) to AdS+N > AAHI-SC2 | " | 0.0249 |
| " | Beta | Untx to AAHI-SC2 (Ho) | " | 0.0004 |
| " | " | Untx to AdS+N > AAHI-SC2 | " | 0.0206 |
| " | " | Untx to AAHI-SC2 > AdS+N | " | 0.0001 |
| " | " | AdS+N (Ho) to AAHI-SC2 (Ho) | " | 0.0002 |
| " | " | AdS+N (Ho) to AdS+N > AAHI-SC2 | " | 0.0198 |
| " | " | AdS+N (Ho) to AAHI-SC2 > AdS+N | " | <0.0001 |
| " | Delta | Untx to AAHI-SC2 (Ho) | " | <0.0001 |
| " | " | Untx to AdS+N > AAHI-SC2 | " | <0.0001 |
| " | " | Untx to AAHI-SC2 > AdS+N | " | <0.0001 |
| " | " | AdS+N (Ho) to AAHI-SC2 (Ho) | " | <0.0001 |
| " | " | AdS+N (Ho) to AdS+N > AAHI-SC2 | " | <0.0001 |
| " | " | AdS+N (Ho) to AAHI-SC2 > AdS+N | " | <0.0001 |
| " | " | AAHI-SC2 (Ho) to AdS+N > AAHI-SC2 | " | 0.0415 |
| " | Omicron | Untx to AAHI-SC2 (Ho) | " | 0.0118 |
| " | " | Untx to AAHI-SC2 > AdS+N | " | 0.0369 |
| " | " | AdS+N (Ho) to AAHI-SC2 (Ho) | " | 0.001 |
| " | " | AdS+N (Ho) to AAHI-SC2 > AdS+N | " | 0.0042 |
| " | " | AAHI-SC2 (Ho) to AdS+N > AAHI-SC2 | " | 0.0065 |
| " | " | AdS+N > AAHI-SC2 to AAHI-SC2 > AdS+N | " | 0.0291 |
| Fig. 8B | AdS+N > AAHI-SC2 | Wuhan to Omicron | " | 0.0002 |
| " | " | Delta to Omicron | " | 0.0113 |
| " | AAHI-SC2 > AdS+N | Wuhan to Delta | " | 0.0508 |
| " | " | Wuhan to Omicron | " | 0.0052 |
| Untx - untreated; Ho - homologous | | | | |
